# The obesity paradigm and the role of health services in obesity prevention: a grounded theory approach

**DOI:** 10.1186/s12913-021-06089-w

**Published:** 2021-02-02

**Authors:** Claire Pearce, Lucie Rychetnik, Andrew Wilson

**Affiliations:** 1 Canberra Health Services, Canberra, Australia; 2 The Australian Prevention Partnership Centre, Sydney, Australia; 3 Menzies Centre for Health Policy, University of Sydney, Sydney, Australia; 4 School of Public Health, Faculty of Medicine and Health, University of Sydney, Sydney, Australia

**Keywords:** Obesity, Prevention, Health services, Systems, Grounded theory, Stigma

## Abstract

**Background:**

Health services have a clear role in the treatment of obesity and diseases linked to obesity but a less well-established role in prevention, particularly in hospital and community-based health services.

**Methods:**

The aim of this research was to examine whether and how hospital and community-based health services incorporate adult obesity prevention into policy and practice. The case study setting was an Australian based health service. Grounded theory informed all aspects of the research including participant recruitment, data collection and data analysis. A systems approach guided the analysis of diverse perspectives, relationships and interconnections within the study context.

**Results:**

The prevailing paradigm within the health service is that obesity is a matter of choice. This dominant perspective combined with a disease focused medical model overly simplifies the complex issue of obesity and reinforces the paradigm which treats obesity as a matter of individual responsibility. A focus on individual change hinders health services from playing an effective role in obesity prevention and leads to unintended consequences, including increasing stigma.

**Conclusions:**

Health service responses to obesity and its prevention compound the negative elements associated with obesity for individuals and are ineffective in creating positive change at individual or a societal level. An alternative systems-level approach is needed to align health service responses with contemporary approaches that address obesity prevention as a complex problem.

## Background

Health systems are faced with the growing impact of non-communicable diseases, particularly those related to lifestyle factors [1]. As life expectancy increases, people are more likely to be living with one or more chronic conditions [2, 3]. In Australia, chronic disease is the major cause of death and disability, with rates highest amongst socially disadvantaged groups [4].

The increasing prevalence of obesity has a strong association with the growing rates of chronic disease [5]. In Australia, approximately two-thirds of adults are overweight or obese and the rate has steadily increased over the past twenty years [6]. Obesity can lead to heart disease, cancer, kidney failure and diabetes as well as being linked to reduced productivity and higher healthcare costs [6, 7]. Being overweight can also impede the management of chronic conditions; it is the second highest risk factor contributing to burden of disease and reduced quality-adjusted life expectancy [8]. The risk of death associated with obesity increases with age and body mass index, with the estimated years of life lost greatest in obese younger adults [9, 10]. For the individual with obesity, carrying excess weight can lead to physical impairment, psychological issues and a reduction in overall quality of life [11].

Health services have a clear role in the treatment of obesity [12], but the role of prevention, particularly in hospital and community-based health services, is less well established [13]. As well as practical issues such as time and resourcing, an important barrier to prevention being incorporated into clinical practice is the perceptions of health service staff regarding obesity and prevention [14]. In this paper we report on a qualitative study conducted within an Australian health service to further explore the views of health service staff from executive, management and clinical levels on whether and how hospital and community health services do or should incorporate adult obesity prevention into their clinical policy and practice.

## Methods

This case study based research drew on the methods of grounded theory, which informed all aspects of the research including participant recruitment, data collection and data analysis [15–17]. The use of a health service setting as a case study ensured all of the data was derived from context-dependent knowledge, enabling the participants to refer to and draw on direct observations or actions within a specific setting [18]. Ethics approval for this research was granted from ACT Health (ethlr.15.250) and the University of Sydney (2016/122). The study was designed utilising the CORE-Q checklist [19].

### Study setting

The data was collected from one comprehensive Australian health service which provides clinical and governance functions for a population of approximately 420,000 people. Clinical services are provided at several centres and include acute and rehabilitative inpatient and outpatient services and community-based secondary health services delivered in community health centres, walk-in-centres and in people’s homes. There is an adult obesity management service which provides lifestyle change management support and access to bariatric surgery but no specific obesity prevention service. Rates of obesity within the population are generally lower than the rest of Australia but are still a major contributor to the burden of disease. In 2014–2015, 63.5 % of the population’s adults were classified as either overweight or obese. This equates to approximately 114,000 (39.1 %) adults being overweight and 69,800 (23.9 %) in the obese range. These rates are a significant increase from two decades ago when only 22.9 % of adults were overweight and 7.2 % were obese [20].

### Data collection methods

In 2016–2017, a total of 43 semi-structured interviews were conducted by the primary researcher (CP). The study research questions were developed and refined through a series of three steps. Step one was a review of the literature confirming that there is a potential role for health services in the prevention of obesity and highlighted some of the main barriers [14]. Step two consisted of telephone interviews with five senior academics with expertise in the field of obesity prevention, followed by thematic analysis of the interview transcripts to identify themes and patterns without referencing a specific theoretical framework [15]. Step three was an analysis of information gathered from the literature and the expert interviews to develop a semi-structured question guide to be used for the case study interviews (see Appendix 1). Each interview was conducted face-to-face by the researcher and recorded. The recordings were transcribed by an external company and each participant was given the opportunity to check and comment on the transcript. The interviews varied in length from 40-80 min.

### Study participants

Interviews were conducted with staff employed by the health service working at three different levels. The sampling for the case study aimed to include participants who represented the macro (executive), meso (clinical management) and micro (clinical) levels of the health system. In the interests of protecting the anonymity of the participants, an organisational chart has not been included. The sample size for each group was determined by theoretical saturation sampling in that data collection; iterative analysis to refine categories continued until no new categories or explanations of those categories were found [15, 16]. Table 1 summarises the final sample of study participants.

**Table 1:** Sampling of study participants

	Executive	Medical	Allied Health	Nursing	TOTAL
**MACRO**	7	3	-	1	11
**MESO**	-	-	6	6	12
**MICRO**	-	-	17	6	23

Eleven face-to-face interviews were conducted with executive level (macro) staff whose roles focussed on policy, population health or clinical governance. At the meso level, twelve interviews were conducted with staff with a team management responsibility for either single or multidisciplinary teams. Each managed a community team providing clinical services in community centres and/or the person’s home. Six of the interviewees had a nursing background and had management responsibility for teams of registered and enrolled nurses. Five had allied health backgrounds and had direct responsibility for the management of single discipline allied health teams. A sixth allied health manager had overall responsibility for allied health teams. The allied health services represented were podiatry, physiotherapy, social work, occupational therapy and dietetics.

The third group of interviewees consisted of allied health and nursing clinicians based within the teams of the managers interviewed. Twenty-three clinicians participated in interviews. Seventeen had allied health backgrounds including podiatry, social work, occupational therapy, physiotherapy and nutrition. Six were registered nurses. Years of practice ranged from less than one to more than 20 years across a range of clinical, education and clinical specialist roles.

### Data analysis

Each transcribed and anonymised interview was reviewed by CP to provide an initial analysis of the data in relation to the research question. Using the ‘comments’ option in Microsoft Word, notes were made about points of interest and elements that may need further exploration in future interviews. Overall memos of the researcher’s initial observations were tabled at the end of the transcribed interviews. Following a second reading, preliminary codes were added to the transcript in a separate column and an overall memo relating to the interview was written.

This was repeated for 3 to 4 interviews before the researcher used theoretical sampling to cross check the initial codes and begin the process of grouping them together as categories in a second column. At this point, the primary researcher discussed their findings with one of the secondary researchers (LR) to reflect on the coding and development of categories. Further memos were prepared labelling the grouped codes into broader categories and included a more detailed explanation of these categories, including their dimensions and variability among participants. Where appropriate illustrative quotes were extracted, and questions or comments were noted by the researcher e.g. where to think further about the grouping of categories or what needed further examination in the data. Reflective discussion with the secondary researchers continued at regular intervals throughout the data analysis process.

Following the initial coding of the data and the identification of data categories, continued analysis and theoretical sampling was undertaken to determine connections between these categories and to analyse the significance of any similarities and differences across different levels of the system. This identified the concepts which formed the basis of the grounded theory to address the research aim of examining how boundaries, relationships and perspectives may enable or hinder the ability of secondary health services to incorporate adult obesity prevention into practice. An example of the data analysis process can be found at Table 2.

**Table 2:** Example of data analysis in the study

Data		Initial codes	Categories	Main concept
‘I’ve asked that anything that is required to take weight of a patient actually has a visible label on now, so that includes chairs, walking aids, whatever, so the clinicians and patients can visibly see the capacity of that equipment. There were two reasons for that. One, was to try and ensure that the staff were aware of what the weight limits were for equipment because they vary amongst suppliers and different pieces of equipment, and also it’s a big tricky and a bit sensitive to have to say to somebody that they breach the weight limit of a piece of equipment, so we’re hoping there’s things such as chairs in waiting rooms and things that patients would be able to make an informed decision around that kind of thing’.		Sensitivities around telling people they are too heavy for equipment- help people make decisions themselves in relation to equipment, weight limits of standard office chairs not high enough as rates of overweight go up?	Stigma Choice Normalising	Normalising of obesity

## Results

The study results revealed that the perceptions of health service staff about the nature and importance of obesity prevention vary according to whether they work at the macro, meso or micro level of the organisation. These views reflect the current health service responses to obesity and have important implications for what is likely to be achieved in prevention going forward. There are differing beliefs and assumptions about how the system works, and what is needed to make improvements, which in turn impact on attitudes towards what can or cannot be done to incorporate obesity prevention into healthcare. These differences are summar ised in Table 3.

**Table 3:** Differences in perceptions across the health system

		EXECUTIVE: ***Macro***	CLINICAL MANAGERS: ***Meso***	CLINICIANS: ***Micro***
**PURPOSE**		Population health with solution focus	Specific clinical groups with process focus	Individual needs – solution focus on individual systems
**INTER-RELATIONSHIPS**	KEY SOURCE OF FEEDBACK	Population health data	Observations of time and resources required to manage individual patient	Limited interdisciplinary feedback
	ASSUMPTIONS	Primary care will address obesity	Dietitians will weigh people and lead on weight loss to address obesity	Need to have rapport with person before can raise weight
	HAVING THE CONVERSATION	Communication about obesity needs to be at a population level	Obesity is a taboo topic and can only be discussed if the individual patient wants to discuss	Need to have rapport and would only discuss weight with a patient as linked to presenting health issue
**PERSPECTIVES**	CONCERN (IN RELATION TO OBESITY)	Physical demands (extra staff/extra equipment/ increased level of service) of caring for people with obesity in hospital and implications for resources	Managing practical issues relating to caring for people with obesity e.g. equipment, double handed visits	Not being able to do enough to address breadth of reasons for why an individual may be obese plus obesity impacting on effectiveness of treatment for presenting problem
	ROLE OF HEALTH IN PREVENTION	Individual prevention is futile, need population approach	Health theoretically has a prevention role BUT too busy providing treatment	Try to do opportunistic prevention with patients – feel they have duty of care
	WEIGHING PATIENTS	Clinicians should weigh patients	Have ensured bariatric weighing equipment is available but do not consider impact of weight in planning of services	Do not routinely weigh patients and many feel shouldn’t weigh patients
**BOUNDARIES**	WHAT LIMITS PREVENTION	Political will to make large scale and legislative changes	Complexity of patients	Service criteria/scope of practice
	POLICY	Responsible for policy – focus on population level and health staff	Aware of policy but it does not impact on the service criteria/clinical focus	Unaware of policy to direct clinical care but are looking for guidance
	CLINICAL FOCUS	Moving healthcare from hospital to community/ reducing hospital demand	Providing services that can manage complex needs	Providing care within service criteria and scope of practice (obesity prevention not in any service criteria)

### Organisational level - senior executives

The macro level staff talked about obesity in general terms, focussing on the overall impact on the population, the link between obesity and chronic disease, and what a population health response should look like. The overall observation from the executive interviews was that while health services can have a role in providing information or education and do take a lead in the treatment of obesity or associated chronic disease, prevention should be led by primary care, with the actual responsibility for taking action sitting with the individual.

‘*At the end of the day, who has the responsibility? The individual does. ……I think the role of health services in preventing obesity is to point out the things that can go wrong’ Executive 6.*

In discussing the impact of obesity on health services, the executive level focused on the idea that being obese significantly increases the risk of an individual developing chronic disease, which has a direct impact on health service resources. This impact on resources results from the increase in demand for services, as well as the extra physical requirements of caring for individuals with obesity, including the need for specialised equipment. The opportunity to incorporate prevention into clinical care was being impeded the by funding systems rather than funding availability, with a lack of financial or other incentives for prevention meaning staff are not motivated to change practices.

*‘Every system is designed to get the results. Every system gets the results that it is designed to deliver. Our system is designed for illness, it is not designed for health.’ Executive 10.*

### Management level - service delivery managers

The meso level managers framed their responses in terms of the caseload their service was responsible for rather than a population-wide narrative, highlighting that their immediate priority as clinical team managers is to provide treatment for discrete groups of patients. They focused on the practical issues of caring for people with obesity within their services, including the requirement for specialist equipment, the physical demands placed on staff and the extra challenges presented when care is being delivered in the home environment.

*‘I think everyone’s feeling very pressured in a day, that they’re trying to get in and out and so they’re sticking to the absolute referral needs rather than unpacking further what’s going on’ Clinical Team Manager 4.*

The pervading view from the clinical managers was that obesity is an issue for community-based services but there is not capacity to undertake obesity prevention as the clinicians are too busy providing treatment for their patients’ immediate health needs. With an ever-increasing demand for health services, particularly as the community caseload takes on more clinically complex issues, treatment rather than prevention must be the focus. The managers articulated that generally by the time people require community health services, many already have weight-related complications, the treatment of which they acknowledged requires a focused, multidisciplinary approach. However, all the community services are single discipline and other than a limited dietetics service, none of the community teams specifically targets obesity.

*‘…. the health dollar is stretched enormously and years and years ago, community nursing would do things like ... we had walking groups and we’d take people walking around the lake…. Those days are long gone. As service demand has increased, those things have fallen away. To suggest that that could be recaptured, is I think a good idea, but probably somewhat idealistic……’ Team Manager 2.*

### Clinical level- clinicians

Across the micro level allied health and nursing groups, the clinicians tended to use an ‘individual care’ narrative by illustrating their answers with de-identified stories relating to patients. The clinicians used this approach to highlight why health services should be taking more responsibility for obesity prevention. They expressed empathy towards people who have obesity and some articulated how they try to incorporate opportunistic prevention into treatment despite an absence of clear policy direction. This lack of guidance resulted in a sense of helplessness as the clinicians want to help their patients, but they don’t really know how.

*‘…a lot of the stuff that you see in terms of the broader, high level stuff that comes from government, it just seems disconnected from the day to day experience of somebody with obesity’ Allied Health Clinician 8.*

The clinicians were highly mindful of the impact of stigma on their patients, particularly in terms of raising weight as an issue. They were also very aware of the stigma that is connected to obesity at a societal level. They could see how obesity links to a person’s self-esteem and that being overweight or feeling unable to address weight can lead to a sense of failure for the individual. Clinicians factored in all these elements when thinking about how to address weight with a patient.

*‘It’s such a self-esteem issue, I think, weight. Not for everyone who holds weight but definitely, a lot of people I see, are quite ... They’re not happy within themselves, I suppose. They find it quite difficult to, maybe, talk about it or maybe they’re embarrassed about their weight’ Clinician AH15.*

### Two key concepts grounded in the data

#### The normalisation of obesity

The perspectives and concerns of health service staff were synthesised and articulated as two core concepts. The first of these was the *normalisation of obesity*. As obesity rates have increased, a normalising process has occurred such as services routinely providing bariatric equipment or modifying treatments to accommodate someone’s weight. At a policy level, there has been an increasing focus on population-based behaviour change outside of the health system and not on developing approaches for individual care within the system while at the clinical level, in the absence of policy, services are tightening service criteria with an increasing emphasis on treatment. Overall, obesity has been perceived as a potential hindrance to ‘normal’ care rather than a chronic condition and as being a burden on resources, rather than as an area that should be prioritised for prevention. As more people present with more complex care needs, this normalising has resulted in a sense of futility about the potential value of health services conducting obesity prevention.

#### The obesity discourse

The second core concept identified was that of the prevailing *obesity discourse*. This concept represents the way people within the health service talk about obesity. This concept is influenced by several factors including personal experience, people’s observations of the impact of obesity on health services, professional philosophy, and an internal narrative around how an individual may experience or respond to being obese. This discourse at times saw different participants take opposing positions, particularly in terms of whether obesity is a disease and whether it is a matter of individual responsibility. This was observed to be an attempt by participants to limit the complexity of obesity with an emphasis on a biomedical approach focusing on linear cause and effect. Participants showed awareness of the shame and stigma that is inextricably linked to the societal response to obesity and this impacts on how they might broach the topic of obesity at an individual level. However, this did not extend to them being able to articulate what role health services could play in addressing this stigma.

## Discussion

In March 2019, the Obesity Collective released a report entitled ‘Weighing in: Australia’s growing obesity epidemic’, which highlighted the growing rates of obesity, the impact of obesity on society and the cost of obesity. The report outlined that there has been a growth in the number of people with the highest class of obesity, measured in terms of their body mass index [21]. While reflecting some diversity associated with the levels at which people work, this research demonstrates that the overarching perspective of staff working at macro, meso or micro levels of one health system was that of obesity being framed as a matter of choice. This framing reinforces the idea that these increasing rates of obesity is as a result of people with obesity not taking on board public health messages to eat better and to increase their physical activity and that people with obesity are not motivated to change. However, as highlighted in the report, blaming people is unfair and it doesn’t work and this combined with health services being delivered within a medical model, means health services are not playing an effective role in the prevention of obesity.

For this case study, it was found that the barriers to clinical services providing obesity prevention were the restrictions caused by service criteria, a lack of clarity around the role of health services in obesity prevention and an absence of feedback between the different areas. This builds on previous research which found a dissonance across the health system in terms of the perceptions of obesity [22] and highlighted blame as being at the centre of a lack of clarity around what is entailed in obesity management versus obesity prevention, as well as the limitations of the medical management discourse [23]. The unresolved status of obesity as a disease compounds these practical issues as health services have a propensity to revert to a disease-based treatment approach, reinforcing the assumption that responsibility for prevention sits with the individual.

It has been previously found that a continued focus on the need for individual change contributes to blame and stigma which ultimately does not result in any positive change at an individual or a societal level [24]. Treatment is ‘normal’ for health so services just keep treating problems as they arise rather than looking at ways of stopping the need for treatment [25]. However, as people with obesity tend to access healthcare services more often, and on admission stay longer [26], there is a need to move past the dominant medical models to evolve the way that health services conceptualise and address obesity.

The question of whether or not obesity is a disease remains an ongoing debate [27]. On face value, defining obesity as a disease may assist health services to resolve practical matters such as directing resources to the treatment of obesity. However, the data analysed for this research showed that a focus on a dichotomous view of obesity as a disease reduces the complexity of obesity to a simple problem with two extremes – one of blame (it is not a disease therefore the person has to fix the problem themselves) or one of biology (it is a disease therefore there needs to be a treatment and ideally a cure) [28] which prevents health services from expanding their role beyond obesity treatment. The concept of prevention within this paradigm focuses on an absence of disease [29] and the relationship between patient and healthcare professional is often simple and binary, which does not help when attempting to address the impact that obesity is having on health care resources [30–32].

It is also difficult for health services to move beyond a discourse of choice and responsibility while society continues to blame individuals not just for their own issues but also for the negative impact of their weight on broader society. Even people with obesity tend to apply an individual blame-centred discourse to their own situation, framing their weight gain as being a shortcoming of their own motivation or inability to deal with specific challenges [22]. Framing obesity as a matter of choice is a way to simplify a complex problem [33] but it can also become an ‘excuse’ for inaction as shifting responsibility to the person with obesity reduces the level of responsibility in the wider social and economic system [23, 34–36]. However, individuals do not exist within a vacuum, there are numerous elements which influence weight. It is not helpful to blame people for their weight nor is it fair or realistic to expect that a simple approach will enable them to make the wide and varied adjustments that are needed for people to change their lifestyle [37]. If the dominant narrative continues to be that obesity is bad for individuals and for society, it will only serve to underpin discrimination [24, 38], compounding the negative effects of obesity.

This research demonstrated that clinicians working in health services witness the problems stemming from what they perceive as a choice and personal responsibility paradigm but they are not supported by health service policies or systems to significantly change their practice. Those responsible for the policy level understand the need for a population approach but not how to support prevention at an individual level. There is a clear need to bring these perspectives together so that health services can play a role in challenging the assumptions and negative stereotypes that frame obesity, and to help develop a paradigm which recognises complexity and moves beyond the concept of choice. If health services frame obesity as a social problem, as one of health inequality rather than a disease to be cured, there may be an opportunity to develop a range of responses and principles rather than relying on a one size fits all linear solution.

Prevention and treatment of obesity are equally important. Health services need to take an approach which strikes a balance between respecting diversity in body size whilst still providing appropriate care for people who have obesity and disease [24]. We also need to accept that society has changed and that a huge part of this change has resulted in the environmental and social influences on weight. This will help shift from a paradigm which frames obesity as being the individual’s fault [30], and move towards a model of action which focuses on shared responsibility where change is achieved at a collective level.

The recommended next step in developing the case studies health obesity prevention system is the facilitation of an iterative series of dialogues between the macro, meso and micro levels of the system to begin to identify and implement locally appropriate, concrete solutions that could be embedded into the system. This will highlight the practical blockages that have resulted from the differing perspectives and open up the possibility of applying an approach beyond that of the traditional medical model to the issues facing health services as a result of increasing rates of obesity. A systems approach will provide practical tools to facilitate this process. For example, bringing together representatives from a policy and a clinical delivery space, as well as consumers within the system to develop a causal loop diagram, will not just lay out what happens within the system, but will also identify the linkages and gaps within the relationships and dynamics impacting on the system in order to identify leverage points [39, 40].

## Conclusions

The overall finding of this research was that the way we frame obesity (within health services) as a matter of choice, and deliver services within a medical disease model, prevents health services from playing an effective role in the prevention of obesity. This builds on previous research which found a dissonance across the health system in terms of the perceptions of obesity [22], which highlighted blame as being at the centre of a lack of clarity around obesity management versus obesity prevention and which showed the limitations of a medical model approach [23] which overly simplifies the complex issue of obesity prevention and further reinforces the paradigm of obesity as a matter of individual responsibility.

Yet there is substantive evidence that obesity is not a choice nor is it a sign of failure or weakness [41, 42]. Rather it is a measure of size which for each individual is the result of a complex combination of many factors and influences. As a society we need to move away from a paradigm of blame which defines people by their weight, to stop attributing a range of negative and unrelated characteristics to a number on a scale and to stop viewing obesity as an illness or disease to be cured [32]. The prevailing narrative leads to unintended consequences as the continued focus on the need for individual change contributes to blame and stigma which compounds the negative elements associated with obesity for the individual and ultimately does not result in any positive change at an individual or a societal level.

The opportunity for change at a macro level within health services may sit with bringing together the divergent views of people based within different parts of the system, and to undertake research which tests ways to reframe the obesity paradigm within health service settings. This information could be used to inform the paradigm underpinning the national preventive health strategy. At the micro level of individual healthcare delivery, health services need to provide explicit obesity prevention policies and service criteria to support clinicians working with individual patients and establish clear referral systems with appropriate resourcing. Services need to be flexible enough to work through the multitude of elements reinforcing or contributing to obesity and to work out what each person’s ideal outcomes would be beyond weight loss. This approach would need to be non-linear and recognise that for individuals there is no set way of addressing the factors that have contributed to weight gain to the point of developing health issues. A systems approach to obesity prevention at the meso level would help develop incentives, support structures and positive feedback loops across the system to identify a broader range of ways to incorporate prevention into practice for health professionals and the health system as a whole. It would also provide an opportunity to discuss and challenge the assumptions underlying the obesity discourse and support the system to move to an obesity paradigm where obesity is approached as a shared problem, one which is everybody’s responsibility.

There is now an opportunity for research which tests ways to reframe the obesity paradigm within health service settings. This includes challenging the obesity discourse which currently focuses on the dichotomy of obesity as a disease, thereby reinforcing biases and stigma. The normalising of obesity, and the lack of quantifiable data to fully understand the impact of the normalising process also presents a research opportunity to investigate ways to improve feedback across the health system so that the experience of clinicians and consumers is reflected in decisions made in relation to obesity policy.

### Strengths and limitations

This research employed a qualitative approach, specifically using grounded theory to avoid introducing a preconceived idea or hypothesis but to instead approach the topics of interest with an open mind. Grounded theory requires a methodical approach but also facilitates an intuitive approach to the data, which can elucidate innovative conclusions in relation to the research questions [16, 17, 43]. As with all research methods, there are limitations. It cannot be assumed that the processes observed in one setting will also be observed in another setting [43]. However, the development of substantive theory, when considered within the broader literature, can provide insight and guidance that can be further explored in other settings.

With any qualitative research, and particularly with grounded theory, it is important to consider the role of ‘self’ [17]. In this research, the researcher is a part of the system being studied and as a health professional, will inevitably have personal views on the role of health services in the prevention of obesity. Developing the research questions following a review of the literature and interviews with academic experts was one technique used to mitigate the risk of bias. The use of a secondary researcher reviewing the primary researcher’s coding was also used as a tool to explore alternative interpretations of the data.

The use of a case study also has its advantages and disadvantages. Case studies are useful in situations where the topic is complex and there is a benefit in retaining a real-world perspective [18]. The case study was chosen as the profile of its health services and of its population have similarities to many other populations across Australia. The researcher is based within the health service, providing an opportunity to access staff as participants and policy information as contextual information. However, while there was no indication that this occurred, there was a small risk that participants may have modified what they said to a potential colleague. Conscious examination of other contexts needs to be employed in considering how the results may be generalised or transferred [44].

## Data Availability

The datasets generated and/or analysed during the current study are not publicly available due to the size of the organisation which participated in this study. It is deemed that even when no names are attached to the data, the participants may be able to be identified through the interview transcripts which is the data source for this study. The data may be available from the corresponding author on reasonable request.

## Appendix

**Table 4:** Interview Guide

OBESITY	- From a population point of view, how does overweight and obesity impact on the ACT population? - How do the increasing rates of overweight and obesity across the population impact on health services?
PREVENTION	*-* In your own words, how would you define prevention as applied to chronic disease? - What are the key elements of a prevention system aimed at reducing rates of overweight and obesity?
POLICY	- Describe the ACT policies which incorporate overweight and obesity prevention? - How do these policies link to service delivery within ACT Health?
ROLE	- What do you see as the role of health services in the prevention of overweight and obesity?
BARRIERS	- What are the main barriers to health services being able to incorporate prevention into service delivery? *Potentially prompt with suggestions: time, skill, embarrassment, not knowing what to do with information*
DATA – KPIS	- What data does ACT Health gather in relation to overweight and obesity? - How does ACT Health measure what is being done to prevent an increase in overweight and obesity?
SKILLS	- Are health professionals equipped with the necessary skills to incorporate prevention into care?
STIGMA	- What do you think are the impacts of the social stigma attached to overweight and obesity? - How does this (the stigma) impact on the capacity of health services to deliver preventive care? - Do you think patients expect the topic of weight to be raised if it is impacting on their health? - Do you think clinical staff are comfortable raising the topic of weight or lifestyle risk factors with their patients?
- Are there any final comments you would like to make or points you would like to raise? - The interview will be transcribed, and I will check for initial errors. Would you like me to send the transcript to you to allow you to check for accuracy? - Are you happy for me to contact you if I require clarification of any of the topics we have discussed today? Is email the best way to contact you?	

## Abbreviation

ACT: Australian Capital Territory
